# Spongy Gums

**Published:** 1859-03

**Authors:** T. W. Fry

**Affiliations:** Crawfordsville, Ind.


ARTICLE V.

### SPONGY GUMS.

BY T. W. FRY, M.D., OF CRAWFORDSVILLE, IND.

On the 28th day of May, 1855, I was called to Fountain County, in consultation with Dr. Thomas, to see Mrs. W., who had been diseased about fourteen years, and had received treatment from a number of physicians, without the slightest improvement. I found her very much emaciated, desponding, and having but little hope of recovery. She was tall, spare, of dark complexion, and evidently of scrofulous diathesis; most of her teeth were badly decayed, her gums were exceedingly spongy, and grown both above and below, so as to envelope the teeth, presenting a perpetually-bleeding surface; she could take food only in a liquid form, and had but little relish for that. From all that I could learn of the treatment previously adopted, it consisted in making incisions in the gums and applying styptics to the bleeding surface. The constitutional remedies were generally of a depleting character, among which were the various preparations of mercury, which served but to increase the virulence of the disease and diminish the hope of recovery. The course of treatment adopted and pursued after consultation with Dr. Thomas, consisted in cutting away all that portion of the gum which had grown above the natural attachment to the teeth, and applying alternately to the exposed surface the tincture of catechu and a solution of the nitrate of silver, in the proportion of ten grains to the ounce. By this means the bleeding and further growth of the gums were prevented. She was immediately put on the use of tonics, iodide of potassium, a more nourishing diet, and cod liver oil. Under this treatment her health rapidly improved, and now, after the lapse of three years, she is in the enjoyment of good health, and has had no recurrence of the disease.

My object, Mr. Editor, in reporting cases that have occurred so long ago, is to be certain that the cures are permanent. The thought has often struck my mind, that very many of our young physicians, ambitious of appearing in the public journals of the day, and of securing an early and brilliant fame, sometimes report extraordinary cures before a sufficient time has elapsed for them to know whether or not they are permanent. How often has it happened, even with the ablest, the most experienced, and the most skillful of our profession, that they have watched with unceasing care and unflagging interest, cases of peculiar nature and rare occurrence, and fondly hoped that success had crowned their long and anxious efforts, but suddenly some unlooked-for, untoward symptoms appear ; their hopes are dashed in a moment, and the patient passes away when convalescence seemed to have been established. These cases may have appeared in leading journals of the world, and added new lustre to the physician wherever those journals are read. Far better would it be for our profession, based, as it should be, upon principles carefully deduced from well-established facts, that less haste should be observed in the publication of cures effected, especially in chronic cases. Then would the literature of our science be disencumbered of much that is useless, if not really pernicious.

---

